# Calibration-Free Cuffless Blood Pressure Estimation Based on a Population With a Diverse Range of Age and Blood Pressure

**DOI:** 10.3389/fmedt.2021.695356

**Published:** 2021-07-27

**Authors:** Syunsuke Yamanaka, Koji Morikawa, Hiroshi Morita, Ji Young Huh, Osamu Yamamura

**Affiliations:** ^1^Department of Emergency Medicine, General Internal Medicine, University of Fukui Hospital, Fukui, Japan; ^2^Connect Inc., Tokyo, Japan; ^3^Emergency and Critical Care Center, Kobe City Medical Center General Hospital, Kobe, Japan; ^4^Second Department of Internal Medicine, University of Fukui Hospital, Fukui, Japan

**Keywords:** continuous blood pressure, electrocardiogram, pulse wave, wavelet transformation, machine learning, cuffless

## Abstract

This study presents a new blood pressure (BP) estimation algorithm utilizing machine learning (ML). A cuffless device that can measure BP without calibration would be precious for portability, continuous measurement, and comfortability, but unfortunately, it does not currently exist. Conventional BP measurement with a cuff is standard, but this method has various problems like inaccurate BP measurement, poor portability, and painful cuff pressure. To overcome these disadvantages, many researchers have developed cuffless BP estimation devices. However, these devices are not clinically applicable because they require advanced preparation before use, such as calibration, do not follow international standards (81060-1:2007), or have been designed using insufficient data sets. The present study was conducted to combat these issues. We recruited 127 participants and obtained 878 raw datasets. According to international standards, our diverse data set included participants from different age groups with a wide variety of blood pressures. We utilized ML to formulate a BP estimation method that did not require calibration. The present study also conformed to the method required by international standards while calculating the level of error in BP estimation. Two essential methods were applied in this study: (a) grouping the participants into five subsets based on the relationship between the pulse transit time and systolic BP by a support vector machine ensemble with bagging (b) applying the information from the wavelet transformation of the pulse wave and the electrocardiogram to the linear regression BP estimation model for each group. For systolic BP, the standard deviation of error for the proposed BP estimation results with cross-validation was 7.74 mmHg, which was an improvement from 17.05 mmHg, as estimated by the conventional pulse-transit-time-based methods. For diastolic BP, the standard deviation of error was 6.42 mmHg for the proposed BP estimation, which was an improvement from 14.05mmHg. The purpose of the present study was to demonstrate and evaluate the performance of the newly developed BP estimation ML method that meets the international standard for non-invasive sphygmomanometers in a population with a diverse range of age and BP.

## Introduction

Non-invasive blood pressure (BP) measurement with cuff-based devices is widely used, and these devices are necessary for various medical situations (1). However, there are some disadvantages of the cuff-based BP measurement methods: (i) a study showed that three out of 10 home cuff-based BP measurement devices were inaccurate (2); (ii) the measurement is usually intermittent and does not capture all the BP changes occurring throughout the measurement period; (iii) the current cuff-based BP devices are still bulky, and are not portable or practical for daily or long-term use (3, 4); (iv) cuff pressure can be painful for some patients; it can also interrupt their state of rest. The cuff pressure results in difficulty for measuring BP during sleep or everyday life or, even worse, may affect the BP measurement itself (5).

Many researchers have developed cuffless BP estimation devices to overcome these disadvantages, allowing patients to monitor BP continuously (6–8). Pulse transit time (PTT) is the pulse wave propagating time from two separate arterial sites on the same cardiac cycle (9, 10), which usually needs to be examined using a continuous electrocardiogram (ECG) (11). The PTT indirectly depends on blood pressure; the higher the pressure, the faster the PTT (11). This phenomenon has been used for non-invasive BP estimation. However, many conventional cuffless PTT-based BP estimation studies have some drawbacks, divided into four categories: (1) analysis of biased, (2) small datasets, (3) studies with devices that required calibration, and (4) insufficient accuracy as required by international standards. Some studies analyzed biased data that included only young participants with a narrow blood pressure range (12, 13), while others had insufficient participants (13–18). Other studies needed additional advanced preparations such as frequent calibrations (19–21), required additional parameters (22, 23), or used devices that needed to be anchored to the body, resulting in annoyance for some users (24). Besides, some studies either had a wide gap of means and standard deviations from the reference (25, 26), or a low regression coefficient (*R*^2^) (27, 28), resulting in the need for more precise mathematical models (29). Table 1 shows the previous studies for non-invasive cuffless BP estimation. Poon and Zhang's cuffless BP measurement was the only study that had handled a large variety of participants' blood pressures (39 with hypertension), range of age (57 ± 27 years old), and large participant number (85 participants) (21). The estimated SBP and DBP in the Poon and Zhang's method differed from the reference BP by 0.6 ± 9.8 mmHg and 0.9 ± 5.6 mmHg, respectively. However, the BP estimation method in Poon and Zhang's study had major shortcomings to need a calibration procedure for each participant, and the accuracy of BP estimation was not precise.

**Table 1:** Prior non-invasive cuffless blood pressure estimation studies.

**References**	**Source**	**# of participants**	**Range of age**	**Methods**	**MAE SBP**	**MAE DBP**
Gao et al. (30)	PPG	65	22–65	W SVM	5.1 ± 4.3	4.6 ± 4.3
Chen et al. (25)	BCG ECG	51	20–74	AS	9.0 ± 5.6	1.8 ± 1.3
Chan et al. (26)	ECG PPG PTT	/	/	AS	7.5 ± 8.8	4.1 ± 5.6
Ding et al. (13)	PTT PPG	27	21–29	AS	−0.4 ± 5.2	−0.1 ± 4.0
Chen et al. (15, 16)	PTT	23/26	19–60	AS	2.2 ± 6.2	−1.5 ± 6.5
Poon and Zhan (21)	PTT	85	/ (57 ± 27)	AS	0.6 ± 9.8	0.9 ± 5.6

*MAE, mean absolute error; SBP, systolic blood pressure; DBP, diastolic blood pressure; PPG, photoplethysmogram; ECG, electrocardiogram; PTT, pulse transit time; BCG, ballistocardiograph; AS, analytical solution; SVM, support vector regression machine; W, wavelet*.

An international standard has already been formulated for cuffless sphygmomanometers (ISO 81060-1:2007) (31). ISO standard is an international standard that must be met when releasing the cuffless, non-invasive blood pressure estimation model as a medical device to the market in the future. However, to the best of our knowledge, a cuffless BP estimation model that meets the international standard does not currently exist. The purpose of the present study was to demonstrate and evaluate the performance of the newly developed ML method for BP estimation that meets the international standard for non-invasive sphygmomanometers in a population with a diverse range of age and BP.

## Methods

### Participants

We recruited 127 participants (73 males and 54 females) at the University of Fukui Hospital and its affiliated institutions. The study was conducted with the approval of the Research Ethics Committee of the University of Fukui (Approval Number: 20148035). Written informed consent was obtained from all the participants. All participants were asked to fill out a medical form that included sex, date of birth, past medical history, and current medications before measuring participant BP. We excluded those participants who were either pregnant, <18 years old, or had a persistent arrhythmia. We recruited participants with a specified range of BP, as required by a protocol of the International Standard of Non-invasive Sphygmomanometers (ISO 81060-1:2007) (31).

### Experimental System

**Figure 1:**
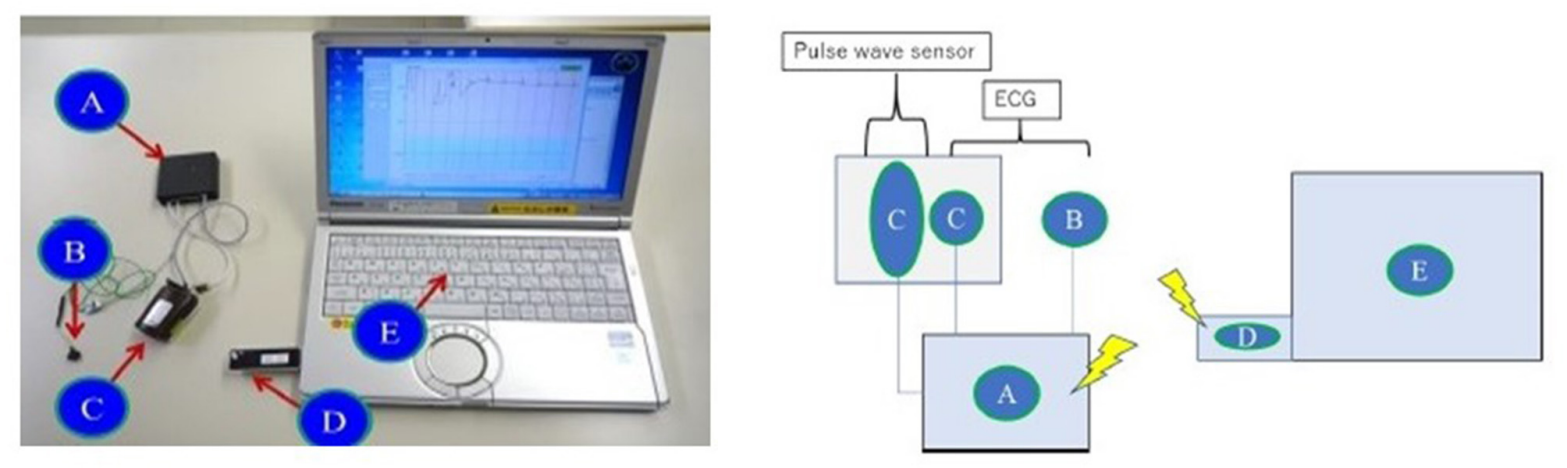
Biopotential sensing system. (A) Biopotential amplification device and data transmitter, (B) First ECG electrical potential electrode, (C) Pulse wave sensor and the second ECG electrical electrode, (D) Receiving dongle, (E) Personal computer for data recording.

A biopotential sensing system was developed to measure ECG and pulse wave simultaneously, as shown in Figure 1. The system consisted of (A) a biopotential amplification device and data transmitter, (B) an ECG electrical potential electrode, (C) a pulse wave sensor and the second ECG electrical potential electrode, (D) a receiving dongle, and (E) a personal computer for data recording. The personal computer recorded and analyzed the waveform of the ECG and the pulse wave. Participants held one of the two ECG sensor electrodes with their thumb and index finger of the left hand (B in Figure 1). Another sensor electrode that could sense the ECG and the pulse wave simultaneously was attached to the index finger of the right hand in the sitting position (C in Figure 1). The sampling frequencies and the resolution of these waveforms were 1024 Hz and 12 bits, respectively.

### Data Collection Protocol

**Figure 2:**
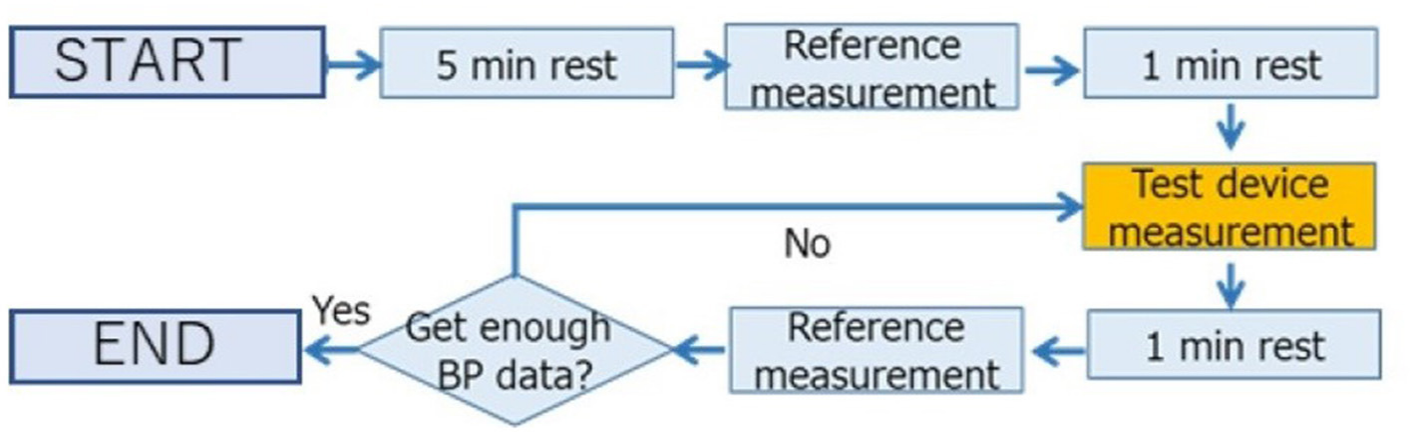
Protocol for collecting data. We took the measurement using the test device in each participant at least three times.

We collected the reference and analyzed datasets according to the protocol of ISO81060-1:2007 (31). We acquired the reference and analyzed datasets from the participants in the sitting position at room temperature without disturbing influences. The left arm of the participants was used for the reference measurement. The protocol for collecting data is shown in Figure 2. After a 5-min rest to stabilize the BP, the first reference BP measurement was taken. Then, the participants took a 1-min rest after the first reference measurement to avoid venous congestion. The measurement was then taken with the device being tested, after the first reference measurement but before the second reference measurement. Participants retook a 1-min rest after the test device data acquisition. After the rest, the second reference measurement was taken. We took the measurement using the test device in each participant at least three times.

#### Reference Data

Two medically trained observers measured the reference BP simultaneously with one reference mercury sphygmomanometer using a “Y” connector that lets two observers measure one participant's BP. The systolic blood pressure (SBP) and diastolic blood pressure (DBP) were determined by phase 1 and phase 5 of Korotkoff sounds, respectively. All measurements were recorded to the nearest 2 mmHg. If the values of SBP and DBP as measured by the two observers were <4 mmHg apart, the mean value of the BP was calculated from the observed values and used as the reference data. If the difference in the measured BP between the two observers was more significant than 4 mmHg, we excluded both the reference and the test device data. These reference BP acquisition methods were stipulated by the ISO standard (31).

### Preparation of the Data Set for Machine Learning

Due to the quality of the measurement, some data were not suitable for data analysis, included either poor ECG, poor pulse wave signals, significant blood pressure changes between the reference measurements taken before and after the test device BP measurement. We applied several structured criteria to the dataset for the preprocessing of machine learning, as shown below:

#### Exclusion Criteria Based on Reference Data

We excluded the data according to exclusion criteria, as designated by the ISO standard for the reference dataset (31); (a) if the difference between the reference measurements taken by the two observers was more than 4 mmHg, (b) if the difference between the reference measurements before and after the test device measurement was more than 12 mmHg for SBP or more than 8 mmHg for DBP, (c) if three valid datasets could not be acquired from one participant due to any reason (e.g., unstable BP).

#### Exclusion Criteria Based on Waveform

We excluded the following cases from the data analysis: (a) the amplitude of the ECG or the pulse waveform was too low due to the dryness of the participant's hands, (b) the crest of the ECG and the pulse waveform was not clear, (c) steady noise (mainly caused due to power supply noise) interfering with the waveform due to unstable electrode holding, and (d) a wavelet calculation error occurring either in the ECG or the pulse waveform mainly caused due to measurement errors. A specific cutoff value was set for each exclusion criteria so that data could be excluded objectively.

#### Data Selection to Satisfy the ISO Protocol

The proportion of participants with high blood pressure and those with low blood pressure was designated according to the protocol of ISO80601-1:2007 (31). ISO requests a wide range of blood pressure proportions to demonstrate the device's applicability to a wide range of participants with various blood pressures. Since we acquired many datasets within the normal range of blood pressure, we needed to exclude some of these datasets from the other datasets for an accurate analysis. The datasets containing large PTT fluctuations and low signal quality of ECG were excluded in descending order until the datasets met the ISO standard protocol.

#### Final Data Set for Training and Evaluation

One hundred and twenty-seven participants with 878 datasets remained in the study. We excluded 21 participants with 423 datasets because of the exclusion criteria based on reference BP data. Nineteen participants with 127 datasets were excluded because of the exclusion criteria based on waveform. Among the 85 patients with 328 datasets, 68 datasets were excluded (66 datasets were normal BP range and 2 datasets were SBP ≥140 mmHg) in descending order of PTT fluctuation and exclude one participant for meeting BP distribution of the ISO standard. Finally, we acquired 260 datasets from 84 participants. The ISO protocol requires more than 85 participants with more than 255 datasets. In this study, we prioritized the distribution of BP values with a certain proportion of participants with high and low BP participants because we aimed to confirm the ability of the device to apply to a wide variety of BP values. The participant's BP distributions are shown in Table 2. We also intended that the device should apply to diverse age groups. The ISO protocol requires the participants' age to range from 18 years old to 65 years old. We collected datasets from young and older participants, and our age range was more expansive than the requirements of the ISO protocols. Table 3 shows the age distribution of the participants.

**Table 2:** Blood pressure distribution of participants.

	**ISO standard**	**Acquired data**
Participant	85	84
Valid data set	255	260
Gender ratio	Each ≥30%	Female 54 (64.3%)
SBP ≤ 100 mmHg	≥13 data/5%	39 data (15.0%)
SBP ≥ 160 mmHg	≥13 data/5%	16 data (6.2%)
SBP ≥ 140 mmHg	≥52 data/20%	52 data (20.0%)
DBP ≤ 60 mmHg	≥13 data/5%	43 data (16.5%)
DBP ≤ 100 mmHg	≥13 data/5%	13 data (5.0%)
DBP ≤ 85 mmHg	≥52 data/20%	71 data (27.3%)

*SBP, systolic blood pressure; DBP, diastolic blood pressure*.

**Table 3:** Age distribution of participants.

**Age (years)**	**58.1 ± 16.1**
20~24	2 (2.4%)
25~29	2 (2.4%)
30~34	5 (6.0%)
35~39	5 (6.0%)
40~44	4 (4.8%)
45~49	5 (6.0%)
50~54	7 (8.3%)
55~59	8 (10.0%)
60~64	13 (15.5%)
65~69	12 (14.3%)
70~74	7 (13.0%)
75~79	7 (13.0%)
80~84	3 (3.6%)
85~	4 (4.8%)

### Implementation of the BP Estimation Algorithm

The proposed algorithm consists of four steps: (i) waveform preparation, (ii) participant group classification, (iii) feature extraction, and (iv) BP value estimation according to the selected participants' group.

#### Waveform Preparation

**Figure 3:**
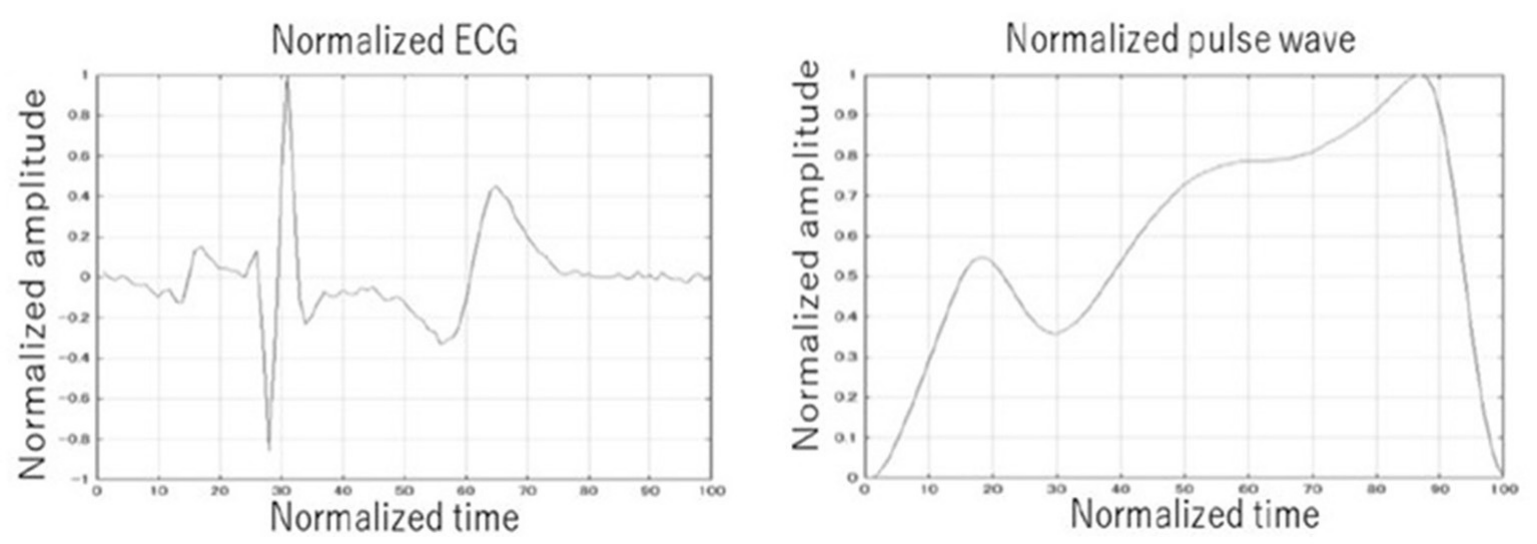
Example of normalized ECG and pulse wave. The ECG and the pulse waveforms were averaged to a single waveform in a 10-second window. The pulse intervals were normalized and extended to one second. Normalization was also applied to the amplitude of the wave.

The ECG and the pulse waveforms were averaged to a single waveform in a 10-s window to decrease the difference between each pulse. Due to the heart rate fluctuations, the pulse intervals were normalized and extended to 1 s. Normalization was also applied to the amplitude of the wave due to the amplitude fluctuation for each pulse. By multiplying the amplitude peak by the multiplication coefficient, the amplitude peak of the waveform is calculated. The R wave peak was defined as the largest peak of the ECG wave from the baseline before and after its generation. As the T wave in ECG is also a big positive wave and we needed to recognize the R wave and the T wave correctly, the threshold was set to 0.7 from the baseline in the normalized amplitude width. The wave that was bigger than 0.7 was recognized as the R wave, and the wave that was smaller than 0.7 just after R wave was recognized as the T wave. The similar procedure was also performed for the pulse waveform. Examples of the normalized ECG and the normalized pulse waveforms are shown in Figure 3.

#### Participants' Group Classification

**Figure 4:**
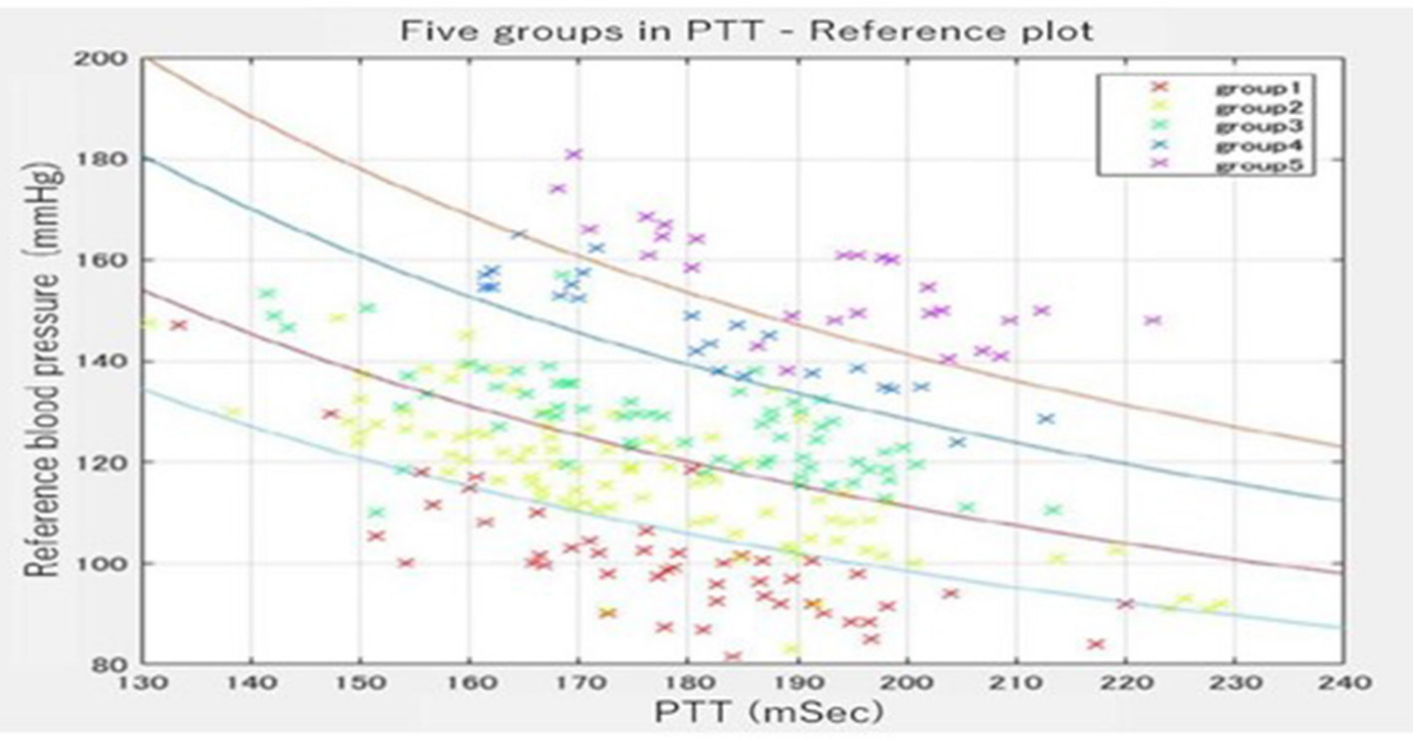
Five groups were divided by group estimator in the PTT-reference plot. The participants were distributed 1:1:3:3:2 in the five areas surrounded by the four curves.

We divided the entire data into five categories (group 1, group 2, group 3, group 4, and group 5.) at a ratio of 1:1:3:3:2 with four curved borders. Figure 4 shows the distribution of sample data divided by the four curved borders. The four curved borders divide the entire data into five subgroups, such as group 1 for the very high BP subgroup, group 2 for the high BP subgroup, group 3 for the moderate BP subgroup, group 4 for the low BP subgroup, and group 5 for the very low BP subgroup at the ratio of 1:1:3:3:2. We noticed that even with almost the same PTT, the BP differed significantly among participants through data collection. However, BP was mainly stable in the same participant. The group classification formula was derived from the relationship between the BP tendency and the waveform features. We expected the classified groups to function as rough BP estimators from waveform features and PTT alone. The ratio of the number of samples was determined as 1:1:3:3:2 while shifting the coefficient term of the 1/PTT line for SBP and PTT. Since we drew four curved borders evenly spaced, subgroup 1(very high BP subgroup) contains very few participants. Hence, we drew the bottom three curves evenly spaced and the top curved border 50% nearly to the second one. As a result, the participants were distributed 1:1:3:3:2 in the five areas surrounded by the four curves.

A support vector machine ensemble with bagging was selected as a group estimator (32, 33). The support vector machine is one machine learning and constructs a hyperplane or set of hyperplanes in a high- or infinite-dimensional space, which can be used for classification, regression, or other tasks like outlier detection. We applied 10-fold cross-validation for learning. Cross-validation is a technique for assessing how the results of a statistical analysis generalize to an independent data set (34). Even the support vector machine (SVM) has been proposed to provide a good generalization performance; if we use only one SVM, the classification result of the practically implemented SVM is often far from the theoretically expected level. To improve the limited classification performance of the one SVM estimator, we prepared 35 support vector machine estimators with bagging (bootstrap technique). Each SVM was trained independently using the randomly chosen training samples via a bootstrap technique. Then, they were aggregated into to make a collective decision for participants' group clarification (32, 33).

#### Feature Extraction

**Figure 5:**
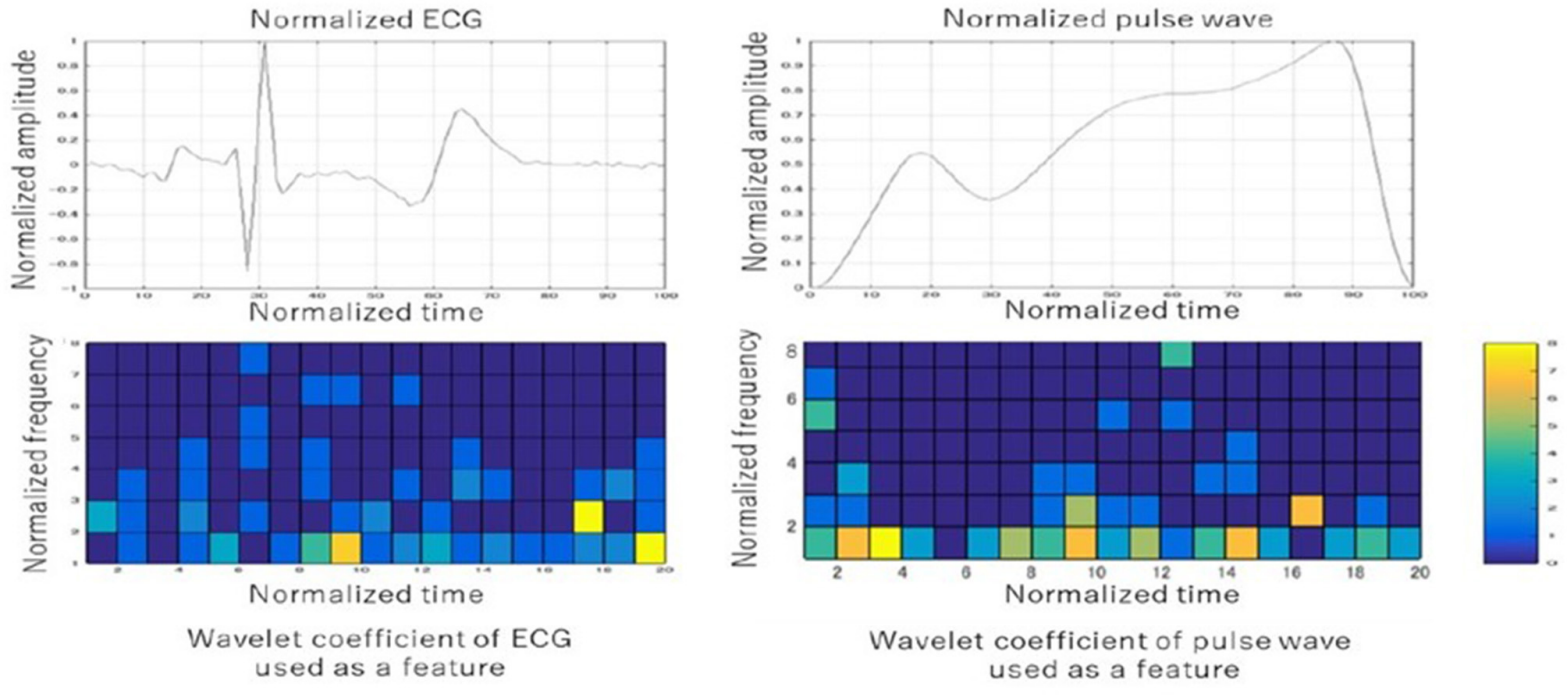
Examples of the wavelet transformation of the ECG and pulse waveforms. The ST-segment and the baseline between the T wave and the P wave had frequently used the wavelet coefficient features in both the wavelet coefficients of the ECG and pulse waveforms.

The candidate features for machine learning consisted of the following features: participants basic information (age, body weight, height, etc.), PTT, basic information of second derivative photoplethysmogram (a, b, c, etc.), wavelet features from the ECG, and wavelet features from the pulse wave. The continuous wavelet transform was adopted for waveform analysis. We obtained the wavelet features from each cell by dividing the normalized wave of both the ECG and the pulse wave into 20 bands in the horizontal and eight bands in the vertical direction. In this way, we prepared 320 features (20 [the horizontal direction] × 8[the vertical direction] × 2[ECG, pulse wave]) as wavelet features from the ECG and the pulse wave. These features were used to create a BP estimation linear regression model for each group. Table 4 shows the specific features that were used in machine learning in descending order. Although some traditional features such as 1/PTT, the square root of body weight, heart rate, PTT, and the peak of ECG were used, many newly-developed features such as wavelet coefficients of the ECG/pulse waveforms were also used in the models. The ST-segment and the baseline between the T wave and the P wave had frequently used the wavelet coefficient features in both the wavelet coefficients of the ECG and pulse waveforms. Figure 5 shows one example of the wavelet transformation of the ECG and pulse waveforms.

**Table 4:** Selected feature values.

**Selected features**	**Waveform**	Normalized time^**^	Normalized frequency^**^
1^*^ 1/ PTT	-	-	-
2. The square root of body weight	-	-	-
3. Wavelet coefficients	Pulse wave	11	7
4. Wavelet coefficients	ECG	14	3
5. Bodyweight	-	-	-
6. Wavelet coefficients	Pulse wave	19	1
7. Heart rate	-	-	-
8. Wavelet coefficients	Pulse wave	1	2
9. Wavelet coefficients	ECG	1	2
10. Wavelet coefficients	Pulse wave	17	1
11. Pulse transit time	-	-	-
12. Wavelet coefficients	ECG	19	2
13. Wavelet coefficients	Pulse wave	13	1
14. Wavelet coefficients	Pulse wave	14	1
15. Wavelet coefficients	Pulse wave	20	1
16. Wavelet coefficients	Pulse wave	5	1
17. Wavelet coefficients	ECG	5	1
18. Wavelet coefficients	ECG	8	1
19. Wavelet coefficients	ECG	19	1
20. Peak of ECG	ECG	-	-
21. Wavelet coefficients	ECG	20	4
22. Wavelet coefficients	Pulse wave	10	1
23. Wavelet coefficients	Pulse wave	8	1
24. Wavelet coefficients	Pulse wave	8	2

*PTT, pulse transit time, ECG, electrocardiogram*.

* *Numbers are assigned in order of the effectiveness of features*.

** *Wavelet coefficients of ECG/pulse wave: The time indicates the number from the beginning of the normalized time axis divided into 20 equal parts. The frequency indicates the number from the beginning of the normalized frequency axis divided into 8 equal parts*.

#### BP Value Estimation From the Selected Participants' Group

The present study's model comprises two steps: grouping estimation by the SVM ensemble with bagging (mentioned above) and the BP estimation linear regression model for each group. The estimation formula for BP value was derived from the relationship between the feature values and the reference BP values. Since the BP estimation model was prepared for each group, five BP estimation models were learned from the 5-grouped datasets. Linear regression was used for the BP value estimator. The incremental feature value selection was applied to select useful features only. Feature value candidates consisted of the wavelet transformation of the ECG and the pulse waveforms, weight, height, body mass index (BMI), PTT, and 1/PTT.

### Primary Outcome

The primary outcome in this study was the evaluation of the models formulated with the standard deviation of the error between the estimated BP value and the reference value. The 10-fold cross-validation was adopted for evaluating the model performance. The original sample was partitioned into 10 subsamples. Of the 10 subsamples, a single subsample was retrained as the validation data for testing the model, and the remaining nine subsamples were used as training data. The cross-validation process was repeated 10 times, with each of the 10 subsamples used exactly once as the validation data. The 10 standard deviation error results were averaged for the evaluation result (34).

## Results

### Comparison of Conventional PTT-Based Method and the Proposed Method

**Figure 6:**
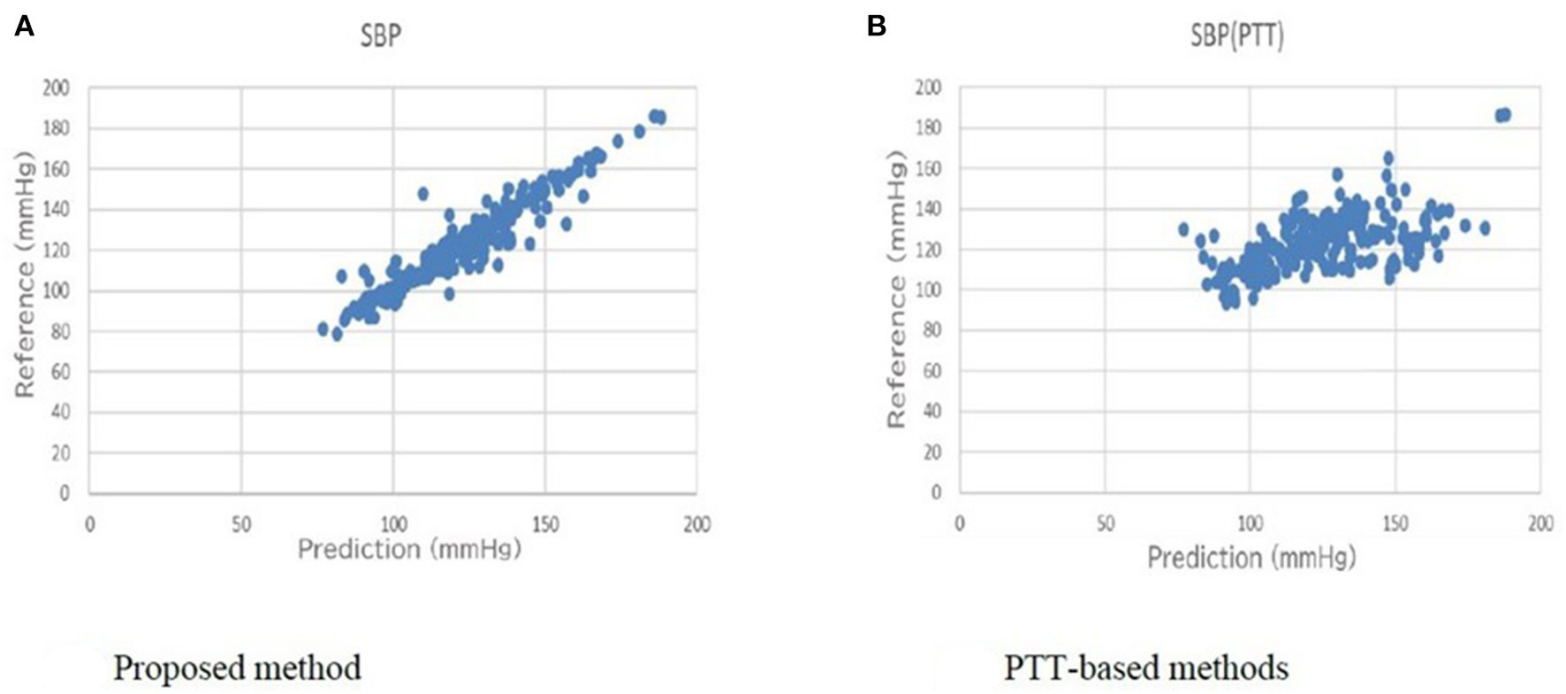
**(A)** Proposed method reference-prediction plot (Systolic blood pressure). **(B)** PTT-based method reference-prediction plot (Systolic blood pressure). The proposed method had small-range prediction values closer to the reference values compared to the PTT-based methods.

**Figure 7:**
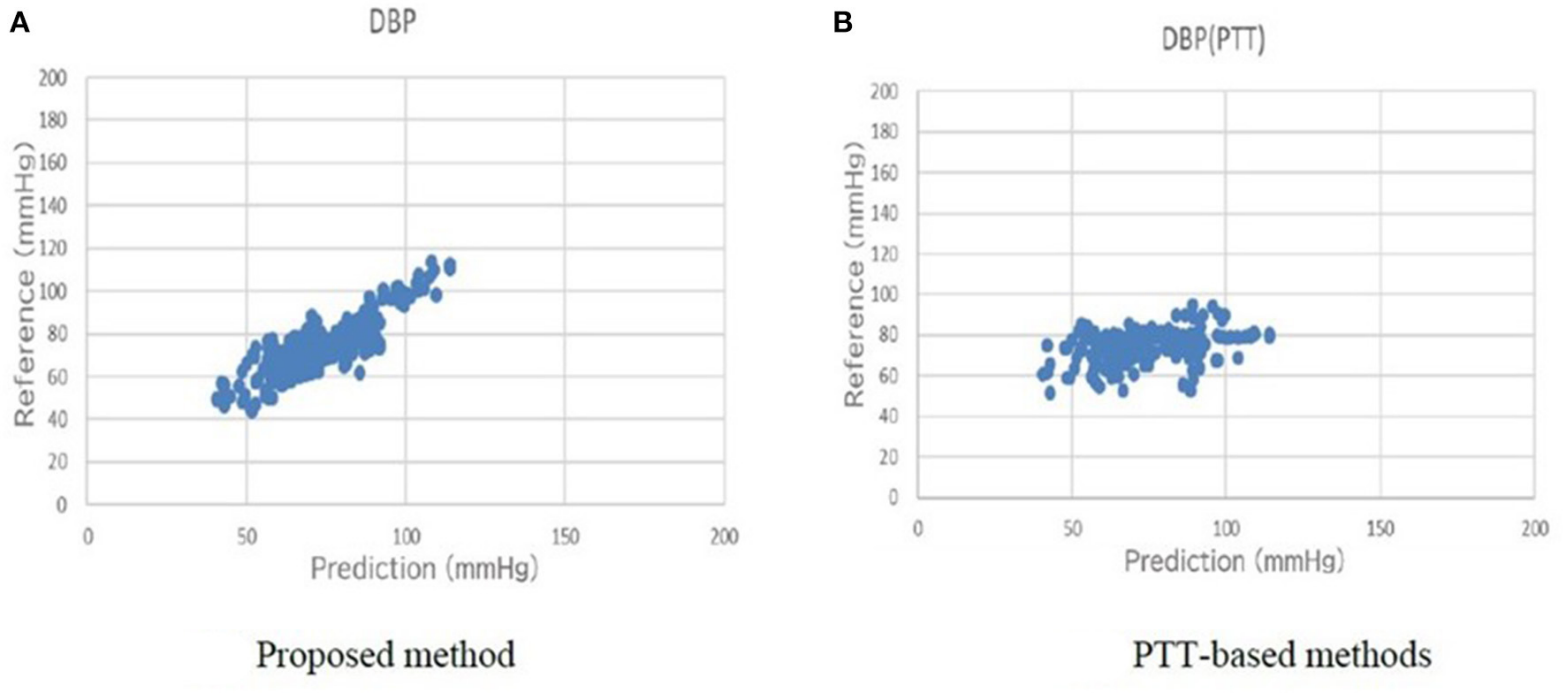
**(A)** Proposed method reference-prediction plot (Diastolic blood pressure). **(B)** PTT-based method reference-prediction plot (Diastolic blood pressure). The proposed method had small-range prediction values closer to the reference values compared to the PTT-based methods.

Table 5 shows a comparison of the conventional PTT-based methods and the proposed method for the primary outcome. For SBP, the error of the standard deviation of the proposed BP estimation results with cross-validation was 7.74 mmHg, which was an improvement from 17.05 mmHg, as estimated by the conventional PTT-based methods. For DBP, the error of the standard deviation of the proposed BP estimation results with cross-validation was 6.42 mmHg, which was an improvement from 14.05 mmHg, as estimated by the conventional PTT-based methods. The association between the estimated BP by the proposed method and the measured reference BP for SBP and DBP are shown in Figures 6, 7, respectively. The proposed method had small-range prediction values closer to the reference values compared to the PTT-based methods.

**Table 5:** Comparison of conventional PTT-based methods and the proposed method.

	**Standard deviation of error in SBP**	**Standard deviation of error in DBP**
^*^Measured BP	21.34 mmHg	14.65 mmHg
PTT-based methods	17.05 mmHg	14.05 mmHg
Proposed method	7.74 mmHg	6.42 mmHg

*BP, blood pressure; SBP, systolic blood pressure; DBP, diastolic blood pressure; PTT, pulse transit time*.

* *Measured BP is the overall distribution of the BP data without any data treatment*.

## Discussions

### Comparison to Prior Work

This study aimed to propose a new BP estimation method that did not require calibration based on a wide range of age and BP distribution while assessing the participants according to the ISO standard protocol. To the best of our knowledge, the present study is the only one that has met most of the protocols of the ISO standard in reference BP acquire method, the number of participants, BP distribution, and the standard deviation of the error for prediction model (<8 mm SBP). Table 1 shows prior studies performed for BP estimation using PTT and ECG analysis. It presents a comparison of the signal source, number of participants, range of age, estimation methods, and error.

### Implementing Wavelet Transformation, Grouping, and Used Features

As shown in Table 4 and Figure 5, the characteristics of the features used in the present models were divided into two: traditional features and newly developed features. Traditional features such as 1/PTT, the square root of body weight, heart rate, and PTT were well-studied, and they were also effective in improving the accuracy in the present study (29). Besides, newly developed features such as wavelet transformation of the ECG and the pulse waveforms were as effective as traditional features (17, 18). The cells of frequently used wavelet coefficient features in both ECG and pulse wave were the ST-segment and the baseline between the T wave and P wave in the ECG (shown in Figure 5). The ST segment represents the interval between ventricular depolarization and repolarization and pumps blood to blood vessels during the ST segment (35). Since the P wave represents the depolarization of the left and right atria and corresponds to atrial contraction, blood moves from the circulatory system to the heart during the baseline between the T wave and P wave (36). Because the arteriosclerosis of the blood vessel wall plays a significant role in determining blood pressure, it seems to be consistent with the many essential wavelet coefficients in the ST segment and the baseline between the T and P waves.

Another feature of the present study was the grouping of the datasets into 5 subgroups. Since the arterial walls are composed of the proteoglycans, endothelium, elastin, collagen, and smooth muscles in varying quantities depending on individuals and vessel size, grouping participants into five subgroups was considered effective (37). By grouping the datasets, the participants' BP estimation model can be accurately selected for each participant that matches the stiffness of the vessel wall.

### The Merit of the Cuffless Sphygmomanometer

We adopted a data collection and evaluation method in line with the ISO standard for non-invasive sphygmomanometers (31). If the estimation accuracy is sufficiently improved, it is possible to implement a cuffless sphygmomanometer that predicts the trends of BP instead of using cuff-based BP devices in the near future. A cuffless sphygmomanometer has many potential advantages. Considerable fluctuations in BP trends can be used as a warning signal by medical practitioners. Most vital parameters in today's operating rooms are measured continuously, except for BP.

Physicians are always concerned about the deterioration of a patient's vital signs in the emergency departments, especially for BP. When we use the model in actual clinical settings in the future, we can use a watch-type pulse wave sensor for 24/7 monitoring. Blood pressure estimates are calculated within 1 s from pulse wave detection, and we can know blood pressure estimates for each pulse, resulting in improved patient safety. In the intensive care unit, invasive arterial blood pressure monitoring is routinely used to monitor seriously ill patients. For these patients, this is not only painful but, more seriously, can cause life-threatening infections or bleeding. However, since the device is non-invasive, the present BP estimation method has no risk of these complications. As it can record BP changes at every beat, it may also contribute to ensuring patient safety by retrospective investigation when incidents occur in a hospital.

A cuffless sphygmomanometer can bring various benefits to ordinary households as well. BP fluctuations are essential in monitoring systems for older patients, like the ones present in smart homes. By installing a cuffless sphygmomanometer in places where sudden changes in the BP of an older individual are likely to occur, such as in beds, chairs, toilets, and bathrooms, the healthcare provider can respond swiftly to these changes. Accumulation of daily blood pressure data can be utilized in outpatient treatment, leading to the improved prescription of appropriate antihypertensive medications and compliance. Furthermore, machine learning with other parameters can lead to the prediction of sudden events in daily life. Therefore, the development of a cuffless sphygmomanometer is expected to impact an aging society's social security system significantly.

### Limitations of the Present Study

First of all, the present study results were data-dependent, and different datasets might create different BP estimation models. The characteristics used in the present study may differ depending on the datasets. In machine learning, 260 data sets in this study are relatively small, and it is desirable to perform sensitivity analysis with more data. However, to our knowledge, this study is one of the largest studies in the field of non-invasive BP estimation. This study also meets the requirements of ISO standards that demanding more than 255 datasets. We have a plan for a validation study with more participants in ICU/ER settings. Secondly, it can be challenging to interpret the created algorithm. As shown in Figure 5, it is challenging to determine precisely why this frequency during the specific period in the ECG and the pulse waveforms were related to the BP. However, these limitations are generally found in ML, and despite these challenges, applying the ML model to clinical practice is rapidly progressing (38–40). Third, we performed waveform measurements on motionless participants and excluded participants in arrhythmias in the present study. Since the current model averages pulse waveforms, patient's movement and arrhythmias can cause the poor performance of BP estimates. Finally, we did not validate the present model with a new dataset in the settings where BP change can be bigger, such as ICU/ER, further validation in ICU/ER settings is needed in the future.

## Conclusions

Based on the participants with a wide age range and BP distribution, we proposed a novel cuffless BP estimation method by grouping participants and applying wavelet features. The standard deviation of error improved from 17.05 to 7.74 mmHg for SBP and from 14.05 to 6.42 mmHg for DBP compared to the PTT-only estimation methods. We plan to increase the number of datasets in ICU and ER settings and improve the accuracy of the estimation methods in future studies.

## Data Availability Statement

The original contributions generated for the study are included in the article/supplementary material, further inquiries can be directed to the corresponding author.

## Ethics Statement

The studies involving human participants were reviewed and approved by the Research Ethics Committee of the University of Fukui (Approval Number: 20148035). The patients/participants provided their written informed consent to participate in this study.

## Author Contributions

SY, KM, and OY contributed to conception and design of the study. KM organized the database and performed the statistical analysis. SY wrote the first draft of the manuscript. All authors contributed to manuscript revision, read, and approved the submitted version.

## Conflict of Interest

KM is employed Connect Inc. The remaining authors declare that the research was conducted in the absence of any commercial or financial relationships that could be construed as a potential conflict of interest.

## Publisher's Note

All claims expressed in this article are solely those of the authors and do not necessarily represent those of their affiliated organizations, or those of the publisher, the editors and the reviewers. Any product that may be evaluated in this article, or claim that may be made by its manufacturer, is not guaranteed or endorsed by the publisher.

## References

[1] Tzourio C Hanon O Godin O Soumaré A Dufouil C. Impact of home blood pressure monitoring on blood pressure control in older individuals: a French randomized study. J Hypertens. (2017) 35:612–20. 10.1097/HJH.0000000000001191 27984412

[2] Leung AA Nerenberg K Daskalopoulou SS McBrien K Zarnke KB Dasgupta K. Hypertension Canada's 2016 Canadian hypertension education program guidelines for blood pressure measurement, diagnosis, assessment of risk, prevention, and treatment of hypertension. Can J Cardiol. (2016) 32:569–88. 10.1016/j.cjca.2016.02.075 27118291

[3] Parati G Stergiou G O'Brien E Asmar R Beilin L Bilo G. European Society of Hypertension practice guidelines for ambulatory blood pressure monitoring. J Hypertens. (2014) 32:1359–66. 10.1097/HJH.0000000000000221 24886823

[4] O'Brien E Parati G Stergiou G Asmar R Beilin L Bilo G. European Society of Hypertension position paper on ambulatory blood pressure monitoring. J Hypertens. (2013) 31:1731–68. 10.1097/HJH.0b013e328363e964 24029863

[5] Ward M Langton JA. Blood pressure measurement. Cont Educ Anaesthes Crit Care Pain. (2007) 7:122–6. 10.1093/bjaceaccp/mkm022

[6] Kim J Park J Kim K Chee Y Lim Y Park K. Development of a nonintrusive blood pressure estimation system for computer users. Telemed J E Health. (2007) 13:57–64. 10.1089/tmj.2006.0034 17309356

[7] Kim JS Chee YJ Park JW Choi JW Park KS. A new approach for non-intrusive monitoring of blood pressure on a toilet seat. Physiol Meas. (2006) 27:203. 10.1088/0967-3334/27/2/010 16400206

[8] Baek HJ Lee HB Kim JS Choi JM Kim KK Park KS. Nonintrusive biological signal monitoring in a car to evaluate a driver's stress and health state. Telemed e-Health. (2009) 15:182–9. 10.1089/tmj.2008.0090 19292628

[9] Nye ER. The effect of blood pressure alteration on the pulse wave velocity. Br Heart J. (1964) 26:261–5. 10.1136/hrt.26.2.261 14132030 PMC1018116

[10] Steptoe A Smulyan H Gribbin B. Pulse wave velocity and blood pressure change: calibration and applications. Psychophysiology. (1976) 13:488–93. 10.1111/j.1469-8986.1976.tb00866.x 972973

[11] Geddes L Voelz M Babbs C Bourland J Tacker W. Pulse transit time as an indicator of arterial blood pressure. Psychophysiology. (1981) 18:71–4. 10.1111/j.1469-8986.1981.tb01545.x 7465731

[12] Teng XF Zhang YT. An evaluation of a PTT-based method for noninvasive and cuffless estimation of arterial blood pressure. Conf Proc IEEE Eng Med Biol Soc. (2006) 1:6049–52. 10.1109/IEMBS.2006.260823 17946738

[13] Ding X-R Zhang Y-T Liu J Dai W-X Tsang HK. Continuous cuffless blood pressure estimation using pulse transit time and photoplethysmogram intensity ratio. IEEE Trans Biomed Eng. (2016) 63:964–72. 10.1109/TBME.2015.2480679 26415147

[14] Jeong I Wood J Finkelstein J. Using individualized pulse transit time calibration to monitor blood pressure during exercise. Stud Health Technol Inform. (2013) 190:39–41. 10.3233/978-1-61499-276-9-39 23823368

[15] Chen Y Wen C Tao G Bi M. Continuous and noninvasive measurement of systolic and diastolic blood pressure by one mathematical model with the same model parameters and two separate pulse wave velocities. Ann Biomed Eng. (2012) 40:871–82. 10.1007/s10439-011-0467-2 22101758

[16] Chen Y Wen C Tao G Bi M Li G. Continuous and noninvasive blood pressure measurement: a novel modeling methodology of the relationship between blood pressure and pulse wave velocity. Ann Biomed Eng. (2009) 37:2222–33. 10.1007/s10439-009-9759-1 19603270

[17] Lee C Zhang Y. Cuffless and noninvasive estimation of blood pressure based on a wavelet transform approach. In: IEEE EMBS Asian-Pacific Conference on Biomedical Engineering. Kyoto (2003). p. 148-9.

[18] Sahoo A Manimegalai P Thanushkodi K. Wavelet based pulse rate and blood pressure estimation system from ECG and PPG signals. In: International Conference on Computer, Communication and Electrical Technology (ICCCET). Tirunelveli (2011). p. 285-9. 10.1109/ICCCET.2011.5762486

[19] McCarthy B O'Flynn B Mathewson A. An investigation of pulse transit time as a non-invasive blood pressure measurement method. J Phys. (2011) 307:012060. 10.1088/1742-6596/307/1/012060

[20] Cattivelli FS Garudadri H. Noninvasive cuffless estimation of blood pressure from pulse arrival time and heart rate with adaptive calibration. In: Sixth International Workshop on Wearable and Implantable Body Sensor Networks Conference. Berkeley, CA: IEEE (2009). p. 114–9. 10.1109/BSN.2009.35

[21] Poon CC Zhang YT. Cuff-less and noninvasive measurements of arterial blood pressure by pulse transit time. Conf Proc IEEE Eng Med Biol Soc. (2005) 6:5877–80. 10.1109/IEMBS.2005.1615827 17281597

[22] Singh RB Cornelissen G Weydahl A Schwartzkopff O Katinas G Otsuka K. Circadian heart rate and blood pressure variability considered for research and patient care. Int J Cardiol. (2003) 87:9–28; discussion 29–30. 10.1016/S0167-5273(02)00308-X 12468050

[23] Liu Q Yan BP Yu CM Zhang YT Poon CC. Attenuation of systolic blood pressure and pulse transit time hysteresis during exercise and recovery in cardiovascular patients. IEEE Trans Biomed Eng. (2014) 61:346–52. 10.1109/TBME.2013.2286998 24158470

[24] Lobodzinski SS Laks MM. New devices for very long-term ECG monitoring. Cardiol J. (2012) 19:210–4. 10.5603/CJ.2012.0039 22461060

[25] Chen Z Yang X Teo JT Ng SH. Noninvasive monitoring of blood pressure using optical ballistocardiography and photoplethysmograph approaches. In: 35th Annual International Conference of the IEEE Engineering in Medicine and Biology Society (EMBC). Osaka: IEEE (2013). p. 2425–8. 10.1109/EMBC.2013.6610029 24110216

[26] Chan K Hung K Zhang Y. Noninvasive and cuffless measurements of blood pressure for telemedicine. In: Conference Proceedings of the 23rd Annual International Conference of the IEEE Engineering in Medicine and Biology Society. Istanbul (2001). p. 3592–3.

[27] Wong Y Zhang Y. The effects of exercises on the relationship between pulse transit time and arterial blood pressure. Conf Proc IEEE Eng Med Biol Soc. (2005) 2005:5576–8. 10.1109/IEMBS.2005.1615748 17281518

[28] Wong MY-M Pickwell-MacPherson E Zhang Y-T. The acute effects of running on blood pressure estimation using pulse transit time in normotensive subjects. Eur J Appl Physiol. (2009) 107:169–75. 10.1007/s00421-009-1112-8 19543907

[29] Sharma M Barbosa K Ho V Griggs D Ghirmai T Krishnan SK. Cuff-less and continuous blood pressure monitoring: a methodological review. Technologies. (2017) 5:21. 10.3390/technologies5020021

[30] Gao SC Wittek P Zhao L Jiang WJ. Data-driven estimation of blood pressure using photoplethysmographic signals. Annu Int Conf IEEE Eng Med Biol Soc. (2016) 2016:766–9. 10.1109/EMBC.2016.7590814 28324937

[31] Non-invasive sphygmomanometers-Part 1: Requirements and test methods for non automated measurement type. ANSI/AAMI/ISO 81060-1. Association for the Advancement of Medical Instrumentation (2007).

[32] Cortes C Vapnik V. Support-vector networks. Mach Learn. (1995) 20:273–97. 10.1007/BF00994018

[33] Kim H-C Pang S Je H-M Kim D Bang S-Y. Support vector machine ensemble with bagging. In: International Workshop on Support Vector Machines. Niagara Falls: Springer (2002). p. 397–408. 10.1007/3-540-45665-1_31

[34] Fushiki T. Estimation of prediction error by using K-fold cross-validation. Stat Comput. (2011) 21:137–46. 10.1007/s11222-009-9153-8

[35] Kashou AH Basit H Malik A. ST Segment. Treasure Island, FL: StatPearls Publishing LLC (2020). 29083566

[36] Douedi S Douedi H. P wave. Treasure Island, FL: StatPearls Publishing LLC (2020). 31869099

[37] Burton AC. Relation of structure to function of the tissues of the wall of blood vessels. Physiol Rev. (1954) 34:619–42. 10.1152/physrev.1954.34.4.619 13215088

[38] Tunthanathip T Sae-Heng S Oearsakul T Sakarunchai I Kaewborisutsakul A Taweesomboonyat C. Machine learning applications for the prediction of surgical site infection in neurological operations. Neurosurg Focus. (2019) 47:E7. 10.3171/2019.5.FOCUS19241 31370028

[39] Kang SY Cha WC Yoo J Kim T Park JH Yoon H. Predicting 30-day mortality of patients with pneumonia in an emergency department setting using machine-learning models. Clin Exp Emerg Med. (2020) 7:197–205. 10.15441/ceem.19.052 33028063 PMC7550804

[40] Fickling SD Smith AM Pawlowski G Ghosh Hajra S Liu CC Farrell K. Brain vital signs detect concussion-related neurophysiological impairments in ice hockey. Brain. (2019) 142:255–62. 10.1093/brain/awy317 30649205 PMC6351777

